# Genome-Wide Identification of MicroRNAs in Response to Low Nitrate Availability in Maize Leaves and Roots

**DOI:** 10.1371/journal.pone.0028009

**Published:** 2011-11-23

**Authors:** Zhenhua Xu, Sihui Zhong, Xinhai Li, Wenxue Li, Steven J. Rothstein, Shihuang Zhang, Yongmei Bi, Chuanxiao Xie

**Affiliations:** 1 Institute of Crop Science, National Key Facility of Crop Gene Resources and Genetic Improvement, Chinese Academy of Agricultural Sciences, Beijing, People's Republic of China; 2 Department of Molecular and Cellular Biology, University of Guelph, Guelph, Ontario, Canada; American University in Cairo, Egypt

## Abstract

**Background:**

Nitrate is the major source of nitrogen available for many crop plants and is often the limiting factor for plant growth and agricultural productivity especially for maize. Many studies have been done identifying the transcriptome changes under low nitrate conditions. However, the microRNAs (miRNAs) varied under nitrate limiting conditions in maize has not been reported. MiRNAs play important roles in abiotic stress responses and nutrient deprivation.

**Methodology/Principal Findings:**

In this study, we used the SmartArray™ and GeneChip® microarray systems to perform a genome-wide search to detect miRNAs responding to the chronic and transient nitrate limiting conditions in maize. Nine miRNA families (miR164, miR169, miR172, miR397, miR398, miR399, miR408, miR528, and miR827) were identified in leaves, and nine miRNA families (miR160, miR167, miR168, miR169, miR319, miR395, miR399, miR408, and miR528) identified in roots. They were verified by real time stem loop RT-PCR, and some with additional time points of nitrate limitation. The miRNAs identified showed overlapping or unique responses to chronic and transient nitrate limitation, as well as tissue specificity. The potential target genes of these miRNAs in maize were identified. The expression of some of these was examined by qRT-PCR. The potential function of these miRNAs in responding to nitrate limitation is described.

**Conclusions/Significance:**

Genome-wide miRNAs responding to nitrate limiting conditions in maize leaves and roots were identified. This provides an insight into the timing and tissue specificity of the transcriptional regulation to low nitrate availability in maize. The knowledge gained will help understand the important roles miRNAs play in maize responding to a nitrogen limiting environment and eventually develop strategies for the improvement of maize genetics.

## Introduction

Nitrate is one of the major forms of inorganic nitrogen in the biosphere and its availability is the limiting factor for plant growth and agricultural productivity for many crops [1]. However, excessive nitrogen (N) fertilization in intensive agricultural areas has resulted in serious environmental problems [2] including soil acidification [3], and the release of reactive nitrogen into the atmosphere, fresh and marine water ecosystems. A better N balance or lower N input is very important for sustainable agricultural production [4]. Therefore, understanding the biological basis of the response of cereals to low nitrate is crucial for the development of crops that utilize N more efficiently.

MicroRNAs (miRNAs) are small, endogenous RNAs that are regulators of gene expression in plants and animals [5], [6], [7], [8], [9]. The identification and study of small RNAs, including miRNAs and trans-acting small interfering RNAs, have added a layer of complexity to the many pathways that regulate plant development [9] and have an important functional role in abiotic stress responses and nutrient deprivation [10]. And, miRNAs have been known for years to be important for phosphate, sulphate and copper deprivation responses in plants [6], [11], [12], [13], [14]. In Arabidopsis, small RNA deep sequencing associated with nitrate response had been analyzed and the miR393/AFB3 has been defined as a unique N responsive module that controls root system architecture in response to external and internal N availability [1]. A cell-specific regulation on lateral root outgrowth in response to nitrogen limitation mediated by microRNA167 had also been defined in Arabidopsis [15]. Comprehensive real-time polymerase chain reaction profiling and/or small RNA deep sequencing has also been used to reveal the existence of complex small RNA-based regulatory networks mediating plant adaptation to mineral nutrient availability in Arabidopsis [1], [16] and rapeseed [16], [17].

Maize is one of the most important crops worldwide and used for food, animal feed, silage, and industrial products. Furthermore, maize crops typically give high yields due in major measure to the use of large amounts of nitrogen fertilizer, which also contributes to a large release of active nitrogen to the environment. Studies on maize have also contributed significantly to our understanding of plant development and evolution as a genetic model system [18]. More recently, this knowledge has been employed to elucidate the regulatory functions of miRNA genes. A genome-wide computational prediction of maize miRNA genes and their characterization with respect to expression, putative targets, evolution following whole genome duplication, and allelic diversity has been reported [19]. However, information about the way by which miRNA are regulated by abiotic stresses in general and by low nitrate in particular is unavailable for maize.

In this work, we used the SmartArray™ and GeneChip® microarray systems to detect the regulation of miRNAs in maize leaves and roots under either chronic N limiting condition or transient low nitrate availability. The corresponding mature miRNAs along with some predicted target genes have also been analyzed for their expression pattern by real time qRT-PCR. Finally, the analysis and prediction of the miRNAs interaction with target genes was performed. Together these results denote the response and role of miRNAs to nitrate-limiting conditions in maize.

## Results

### miRNAs responsive to chronic low nitrate availability identified in maize leaves and roots

**Figure 1:**
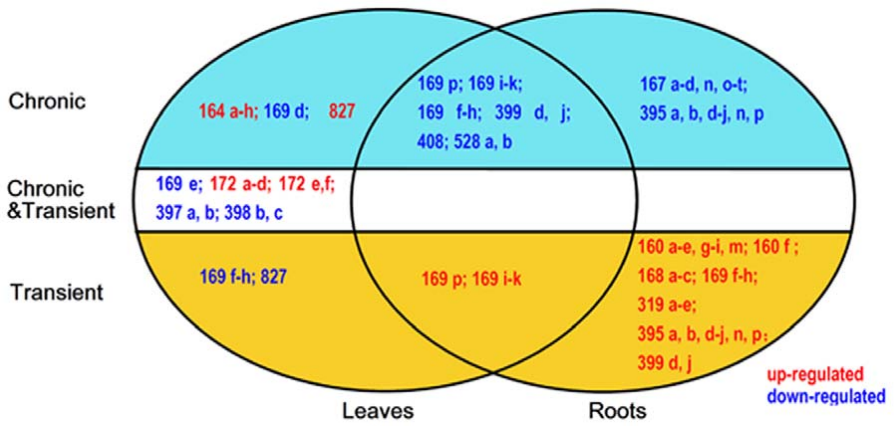
The Chronic (∼15 days) and transient (2 hr) low nitrate regulated mature miRNA families and species identified in leaves and roots. Those regulated miRNAs with fold change>2 or <0.5 and q value<0.001 are shown. Left circle: the responsive miRNAs in leaves; Right circle: the responsive miRNAs in root tips; Light blue shadow: the chronic responsive miRNAs; Yellow shadow: the transient responsive miRNAs; White background: both chronic and transient responsive miRNAs. Words in red: up-regulated miRNAs; Words in blue: down-regulated miRNAs. miRNA families are represented by numbers and their species are represented by the letters.

Maize plants were grown under optimal and nitrogen-limiting conditions for 15 days after germination (details in the Methods section). The total biomass (dry weight) for the plants grown under the optimum nitrate treatment was approximately 2.5 times of those grown under the low nitrate treatment, demonstrating that the low level of nitrate substantively limited growth (Table 1). Leaves and roots were harvested in liquid nitrogen and RNA was extracted immediately for microarray hybridization. The SmartArray™ system was used initially to identify miRNAs responsive to stable N stress. Affymetrix GeneChip® miRNA Arrays system was used later to identify miRNAs responsive to transient N change (see the Methods section). Nine miRNA faimlies (miR164, miR169, miR172, miR397, miR398, miR399, miR408, miR528, and miR827) were identified to be differentially expressed in leaves in response to chronic low N condition. Under N-limiting condition, three of the miRNAs (miR164, miR172, and miR827) were up-regulated while the others were down-regulated (Table S1). Six miRNAs (miR167, miR169, miR395, miR399, miR408, and miR528) were found in roots in response to the chronic low nitrate condition, all of which were down-regulated (Table S2). All of the miRNAs identified in leaves and roots were verified by real time stem loop RT-PCR on mature miRNAs. The probe sets for the different species from each miRNA family, the sequences of the mature miRNAs, and the fold change are listed in Tables S1 and S2. Among these miRNAs, miR169 (169p; 169f,g,h; 169i,j,k), miR399 (399d,j), miR408, and miR528 (528a,b) were found to be N-responsive in both leaves and roots (Fig. 1). Some species in one miRNA family, such as miR169d and miR169e, showed tissue-specific patterns in leaves and roots under N-limiting condition, in that they were down-regulated in leaves but not responsive in roots (Tables S1 and S2; Fig. 1).

**Table 1:** Chronically low nitrate treatment (0.04 mM, 15 days) reduced the biomass and changed the biomass partition in maize seedling (per plant).

NO_3_^−^(mM)	Weight	Tissues	Mean (g)±STD	Root: Shoot	Totalweight (g)
0.04	Fresh	Root	0.94±0.23	0.45	3.02
		Shoot	2.08±0.54		
	Dry	Root	0.07±0.02	0.42	0.25
		Shoot	0.17±0.04		
4	Fresh	Root	1.60±0.90	0.23	8.46
		Shoot	6.85±3.68		
	Dry	Root	0.12±0.07	0.23	0.62
		Shoot	0.51±0.27		

Note: The mean of biomass (fresh or dry weight) along with its partition were determined based on at least 5 biological replicates. The significant level (one pair *t* test) of the difference of biomass (dry weight) between 0.04 mM and 4 mM nitrate treatments is 0.00025.

### miRNAs responsive to the transient low nitrate availability in maize leaves and roots

The genome-wide identification of miRNAs responsive to the transient low N condition was also performed. RNAs were extracted from plants grown under the optimal condition for 15 D and then transferred to low N for 2 hrs. Some selected miRNAs identified from leaf chronic low N experiment were tested for leaf transient low N experiment by the stem-loop RT-PCR method. Five miRNAs (miR169, miR172, miR397, miR398, and miR827) were identified as being differentially expressed in leaves in response to the transient low N condition, with miR172 up-regulated but miR397, miR398, and miR827 down-regulated (Table S1). Interestingly, different species in the miR169 family showed different patterns, as miR169e,f,g,h were down-regulated, but miR169i,j.k,p were up-regulated (Table S1). Six miRNAs (miR160, miR168, miR169, miR319, miR395, and miR399) were identified to be differentially expressed in roots in response to the transient low N condition and all of them were up-regulated (Table S2). These miRNAs were verified by real time stem loop RT-PCR on mature miRNAs (Table S1 and S2). Only miR169 (169p; 169i,j,k) was found to be expressed in both leaves and roots during this transient response (Fig. 1).

**Figure 2:**
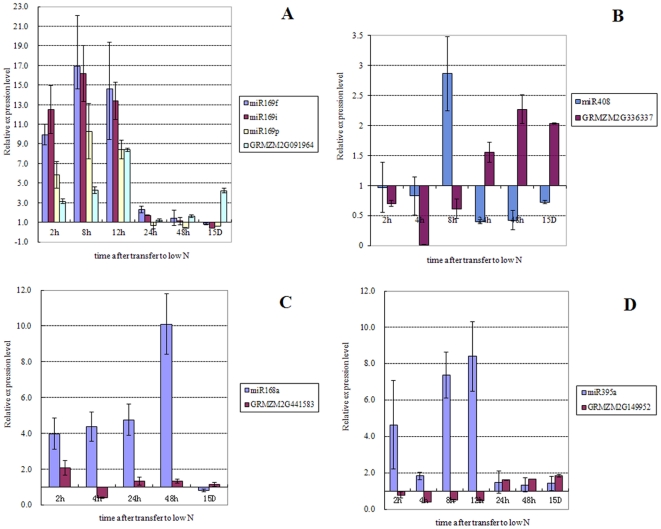
Verification of the low nitrate responsive pattern of the mature miRNAs identified in the roots of maize seedlings and their predicted target genes by qRT-PCR. The X axis is the time of hours (h) after transfer to low N, and the 15D sample is from plants grown under chronic low N. The expression level is expressed as the mean of relative fold changes of triplicate biological replicates and the vertical bars represent standard derivation of the mean (n = 3).

The analysis of the expression of the mature miRNAs showed the consistency of most of the results between the microarray and the qRT-PCR, the only exception was for the chronic expression of miR395 (Fig. 2), with fold-change values of 1.42 and 0.42 for the qRT-PCR and chip hybridization, respectively (Table S2).

### Comparison of the miRNAs responsive to chronic and transient N limitation and the time-course of miRNAs expression under the low nitrate availability

The five miRNAs (miR169, miR172, miR397, miR398, and miR827) identified in leaves in response to transient low N condition were among the nine miRNAs identified under chronic N-limiting condition (Table S1, Fig. 1). Three of these five miRNAs (miR172, miR397, miR398) shared the same expression patterns in their response to both chronic and transient N-limiting conditions, while one of them (miRNA827) showed an inverse response to the two conditions (Table S1, Fig. 1). Within the miR169 family, different species showed different response patterns. miR169e,f,g,h were down-regulated under both chronic and transient N-limiting conditions, while miR169i,j.k,p were up-regulated (Table S1, Fig. 1). Three miRNAs (miR160, miR168, miR319) were identified in roots from the transient response in addition to the chronic response (Table S2). miR169 responded to both chronic and transient N limitation but in an opposite fashion (Table S2, Fig. 1).

**Figure 3:**
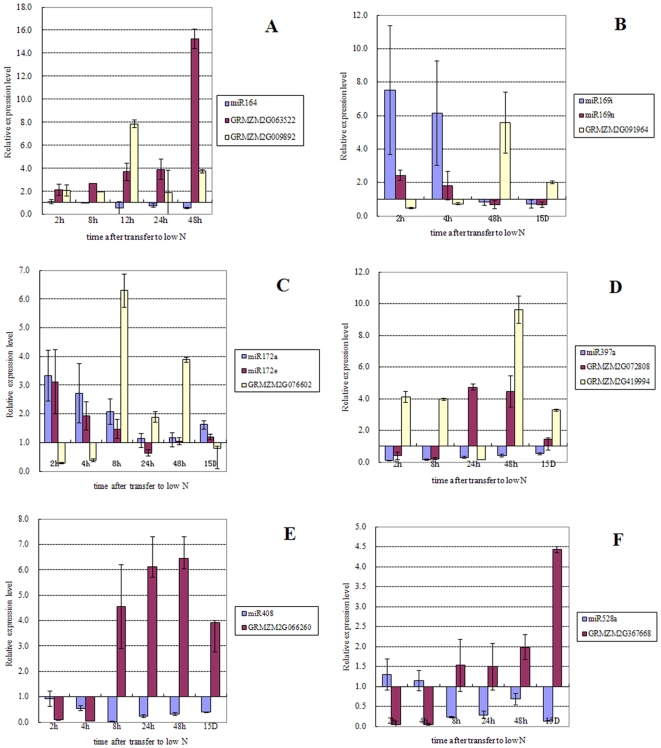
Verification of the low nitrate responsive pattern of the mature miRNAs identified in leaves and their predicted target genes by qRT-PCR. The X axis is the time in hours (h) after transfer to low N, and the 15D sample is from plants grown under chronic low N. The expression level is expressed as the mean of relative fold changes of triplicate biological replicates and the vertical bars represent standard derivation of the mean (n = 3).

In order to examine how the expression patterns of these miRNAs would change at different time points under N limitation, 15-day-old plants grown under optimal N condition were transferred to low N condition for various times ranging from 2 to 48 hrs. The time course expression for some of the miRNAs identified was examined by qRT-PCR, especially for those miRNAs having an opposite trend in their response to chronic and transient N limitation. miR169i,j,k,p had an opposite pattern in both leaves (Table S1, Fig. 1) and roots (Table S2, Fig. 1). The time course results indicate that although miR169i and miR169p (Fig. 2, Fig. 3) were up-regulated at early time points, they were down-regulated after experiencing a longer time of N reduction, suggesting the existence of a possible feedback regulatory mechanism. Most of the miRNAs tested matched the regulation patterns as discovered in the chronic and transient 2 hr array results including miR172a (Fig. 3), miR397a (Fig. 3), miR408 (Fig. 3) in leaves, and miR168a (Fig. 2) in roots.

### Identification of the potential miRNA target genes and their expression profiles in maize

Potential miRNA target genes were identified in maize according to Zhang et al [20], and the results presented in Tables S1 and S2, along with the description of the function of these genes. In order to find possible miRNA/target gene modules in response to low nitrate availability in maize leaves and roots, the expression profiles of some predicted target genes were examined by qRT-PCR. In leaves, miR164 was down- regulated, while its predicted targets, GRMZM2G063522 and GRMZM2G009892, were up-regulated (Fig. 3) as expected, and these code for proteins that are members of the NAC domain super-family (SCOP: 101941). For miR169i and miR169p, the mature miRNAs were up-regulated rapidly after transfer to the low N condition, and then later in the time-course their levels decreased below the level seen prior to N deprivation. The expression level of the predicted target, GRMZM2G091964, showed the expected inverse pattern of expression that would be predicted if it is indeed regulated by miR169 (Fig. 3). GRMZM2G091964 is one of the DNA-dependent CCAAT transcription factors. Both predicted targets of miR397, GRMZM2G072808 and GRMZM2G419994, are putative multi-copper oxidase. However, the latter showed a more sensitive response pattern to nitrogen limitation than the former (Fig. 2). Other multi-copper oxidases that showed a similar pattern to miR397 include miR408/GRMZM2G066260 (Cupredoxin) (Fig. 3) and miR528/GRMZM2G367668 (multi-copper oxidase)(Fig. 3), suggesting that multi-copper oxidase activity involved in electron transport and in oxidase activity might be an important aspect of the physiological response to N limitation. In roots, the expression of miR169f, miR169i and miR169p were up-regulated until 12 hrs, and then returned to the basal level. The RNA expression level of the predicted target, GRMZM2G091964, showed the expected inverse expression pattern (Fig. 2). A similar pattern was seen for miR408/cupredoxin in both roots and leaves (Fig. 2). A microRNA homeostasis module of miR168/ARGONAUT (AGO) (Fig. 2) might be involved in the stress adaptation process. Similarly, the module of miR395/ATP-sulfurylase (Fig. 2) involved in sulphur assimilation showed transient repression at low N.

## Discussion

### miRNAs identified under chronic and transient N-limiting conditions in maize leaves and roots

A number of studies have been done on profiling the transcriptome under various N-limiting conditions in *Arabidopsis thaliana* [15], [21], [22], [23], [24], [25], [26], [27], [28], [29], [30], tomato [31] and rice [32]. However, there has been little information available on the transcriptional regulation to N limitation in maize. Specifically, no N-responsive miRNAs in maize have been identified and analyzed for their potential roles in modulating their expression in response to a N-limiting condition. We used the SmartArray™ and GeneChip® microarray systems and identified miRNAs in maize leaves and roots under both chronic and transient N-limiting conditions. Under chronic limitation, as expected, there is a significant decrease in biomass formation as well as a change in the partitioning of biomass to roots when compared to shoots. The chronic N-limiting condition decreased biomass production by 60%. The direct use of nitrate as the sole nitrogen source, similar to Bi et al (2009) [33], eliminated any possibility of a change in the transcriptome profile triggered by a change in nitrogen source to others like ammonium. The transient N-limiting conditions involved growing plants under optimal N for 15 days and then growing the plants under the low N condition. Based on previous studies where significant transcriptome changes occurred after 2 hours under the low N condition [32], this time-point was chosen to do the initial scan of miRNA expression. A time-course was then done to study the different expression patterns of the miRNAs identified in the microarray analysis.

**Figure 4:**
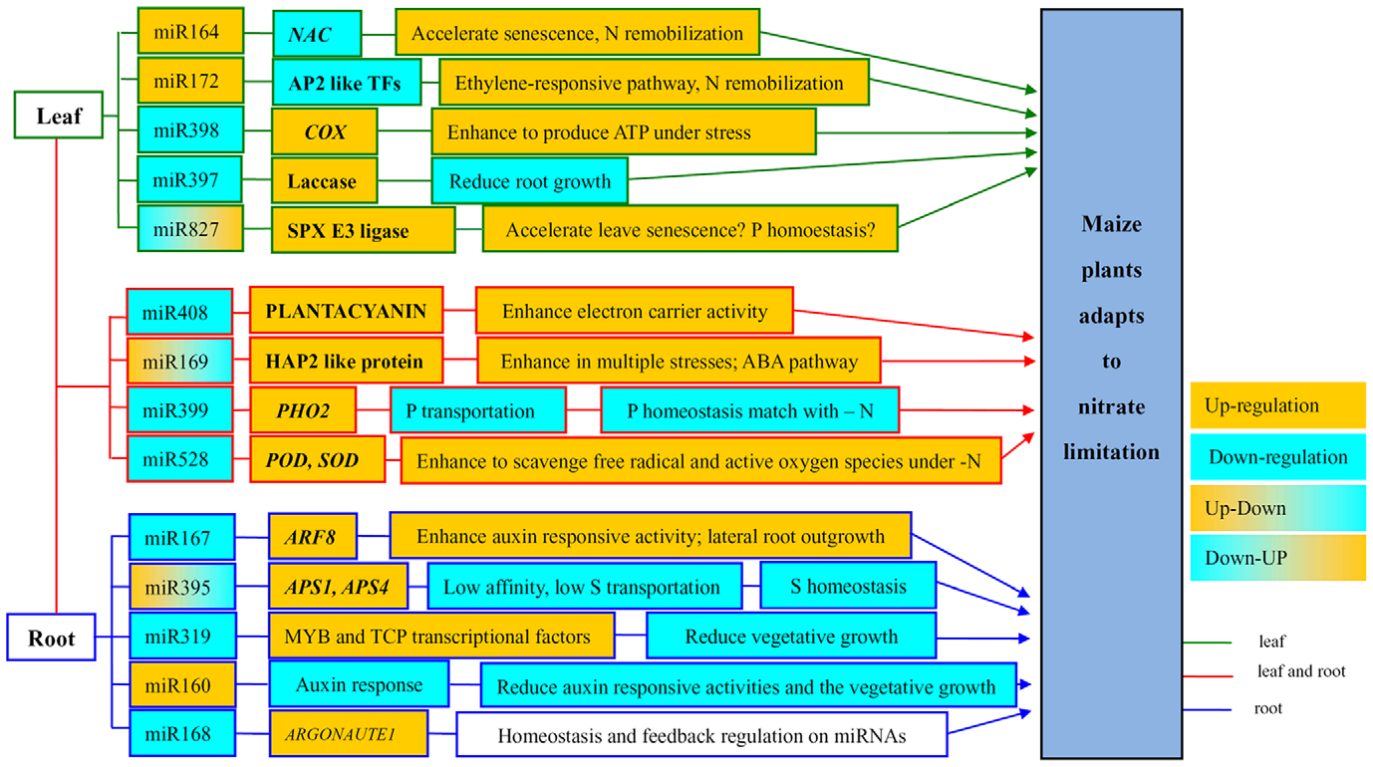
The potential regulatory network for low nitrate responsive miRNAs in maize leaves and roots.

The plant miRNA V2.0 SmartArray™ contains 348 well-characterized and 78 predicted plant miRNAs from various plant species including Arabidopsis, maize, rice, and soybean [34], [35]. The Affymetrix GeneChip® miRNA Arrays contains 6703 miRNA probes from 70 species [36]. Among them, 1631 were characterized and designed based on miRNA (including redundant miRNAs ) from 21 plant species. In total, 2057 plant miRNA probes were presented in the hybridazation. Using these two miRNA array platforms, 14 miRNAs were identified as being regulated under chronic or transient treatments as summarized in Fig. 4, and their expression patterns can be divided into three groups. First, some miRNAs showed differential expression under the various treatments in leaves or roots. For example, miR164 was only up-regulated in leaves under chronic nitrogen limitation. MiR160(a,b,c,d,e,g,h,i,m) was only up-regulated in roots during transient low nitrate treatment. Second, some miRNA have the same response trend to chronic and transient nitrogen-limiting conditions. For example, miR398(b,c), miR172(a,b,c,d) and miR397(a,b) had the same response under chronic and transient treatments in leaves, while miR408 and miR169(f,g,h) were down-regulated in both treatments and both tissues. Third, the majority of regulated miRNAs had different responses to chronic and transient treatments. For example miR169(i,j,k), miR395(a,b,c,d,e,f,g,h,i) and miR169p were up-regulated in the transient condition, but down-regulated during chronic nitrogen stress. For the first and second patterns, the regulation of these miRNAs required a long-term response. For the third pattern, after a certain time where the expression was changed, their concentration returned to or over-shot the basal level. This implies that maize quickly responded to the change in nitrate concentration with regards to the expression of these miRNAs followed by a return to the normal expression level. With regards to tissue specificity(or tissue dependent), some miRNAs were only regulated in roots or leaves, such as miR160, miR167, miR168, miR319 and miR395 in roots, and miR164, miR172, miR397, miR398 and miR827 in leaves, while some others were regulated in both tissues, such as miR169, miR399, miR408 and miR528 (Fig. 4). This is not surprising given that some miRNAs have been shown to accumulate differentially in tissues in both Arabidopsis and rice [37], [38].

### Potential roles of the miRNAs identified during nitrogen limitation

The full list of the predicted target genes of identified miRNAs had been listed in this study (Table S3). According to the function of the target genes (Tables S1 and S2), we can divide the identified miRNAs into three categories. The first includes miR160, miR164, miR167, miR169, miR172, and miR319, which target transcription factors involved in further regulation of gene expression and signal transduction. The eight predicted maize miR160 target genes include seven genes homologous to the *Arabidopopsis ARF10*, *ARF16* and *ARF17* genes. *ARF17* has been found to be essential for embryonic, vegetative, floral and root development, while the *ARF10* and *ARF16* knockout mutants do not show developmental abnormalities [39]. There are 30 miR164 putative target genes in maize which include seven *NAC* transcription factors and three *MYB* domain transcription factors of unknown function, with the rest being involved in diverse processes. The seven *NAC* gene family members are all NAM (no apical meristem) genes which are crucial for meristem development [40]. A quantitative trait locus (QTL) encoding a *NAC* transcription factor, a putative target of miR164, had been shown to accelerate senescence and increase nutrient remobilization from leaves to developing grains in ancient wheat [41]. It implies that zma-miR164 might play a role in remobilizing the nitrogen from old to new leaves to deal with the N-limiting condition. The maize miR167 is predicted to target nine genes including eight homologous to the Arabidopsis *ARF6* and *ARF8* genes. *ARF6* and *ARF8* are associated with the repression of lateral root development during nitrate limitation in Arabidopsis [15]. The targets of miR169s have several *HAP2* transcription factors associated with nutrient deficiency and drought stress [17]. Our experiments showed that the expression of miR169 species had been repressed under low nitrogen and was consistent with the response of the pri-miR169 under low nitrogen treatment in Arabidopsis [17]. MiR172 has eight potential target genes including five *APETALA2* (*AP2*) like transcription factors. In maize, miR172, also known as *tasselseed4 (ts4)*, was shown to be involved in the regulation of maize floral organ identity and meristem acquisition through the target gene which is the *APETALA2* (*AP2*) transcription factor *ids1* [42]. Salvi et al [43] once reported an interesting work on mapping and cloning a flowering time locus of ZmRap2.7, one of *AP2* like orthologs with the target site for miR172, which was therefore likely to be also regulated by an miR172-mediated trans-acting mechanism. MiR319 has eight potential target genes including two *TCP* and two *MYB* transcription factor genes. The *TCP* genes are homologous to the Arabidopsis *TCP2* genes that have been shown to be involved in lateral shoot organ morphogenesis [44] and also help control leaf senescence by regulating jasmonic acid biosynthesis [45].

The second category includes miR395, miR397, miR398, miR399, miR408, miR528, and miR827, whose potential target genes are predominantly involved in energy metabolism and scavenging of the oxidative species produced during stress. MiR395 targets five genes in maize with two being the ATP sulfurylase genes, which catalyze the first step in the sulphur assimilation pathway and are involved in the response to sulphate starvation in Arabidopsis [12]. In addition, miR395 was found to be important for S and P homeostasis in Arabidopsis [46]. It remains to be investigated how miR395 is involved in the crosstalk between N, S, and P nutrient availability. MiR397b has been predicted to target a laccase gene which when mutated was shown to reduce root growth under dehydration [47]. MiR398 and miR408 were down-regulated by Cu/Fe induced oxidative stress to increase *CSD1* (Cu-Zn superoxide dismutase1) and CSD2 level in Arabidopsis [48]. The down-regulation of the copper proteins *COX5b* and the copper superoxide dismutase, *CSD1*, was found under water deficit conditions in *Medicago* [49]. In our case, miR398a/b was up-regulated in both shoots and roots, indicating that miR398 responded differently to different stress conditions. The function of the sole miR398 target gene in maize is unknown. MiR399 is up-regulated by Pi starvation and the target gene *UBC24* (ubiquitin-conjugationg E2 enzyme) is down-regulated in Arabidopsis [50]. The target genes of miR399 in maize, however, are not homologous to *UBC24*. Instead, six out of the 12 potential maize target genes are in the Major Facilitator super-family which are membrane transporters [51]. The module of miR399/*PHO2* had been defined as being involved in Pi signalling and regulating pathways [13]. Given our results, it can be suggested that miR399 might play a role in reducing Pi transport to keep a balance between N and P. MiR528 was found to be repressed in response to drought stress in leaves in *T. dicoccoides* and there are no verified target genes [52]. Rice miR528 had been shown to be down-regulated during the early submergence phase and induced after 24 h of submergence in maize roots [53]. In our case, the stable strong repression of miR528 was found in both roots and shoots under the low N condition. The distinct role of miR528 in multiple stresses needs further investigation. The Arabidopsis miR827 is specifically up-regulated by phosphate deficiency [16], where the expression level of miR827 didn't show significant change to N limitation [37]. Unlike the Arabidopsis and rice miRNA827, we found that miR827 in maize (zma-miR827) showed a significant change in response to both chronic and transient N limitation. This result was confirmed by additional tests (data not shown). In Arabidopsis, the target of miR827 is the Nitrogen Limitation Adaptation gene *AtNLA* involved in the regulation of N limitation adaptation response [54]. It had been demonstrate that *AtNLA* and miR827 have pivotal roles in regulating Pi homeostasis in a nitrate-dependent fashion in Arabidopsis [55]. In maize, there are three potential target genes including a SPX domain protein (GRMZM2G166976), tropomysin and an NADP binding protein. None of these is the *AtNLA* ortholog gene in maize. The characterization of rice osa-miR827 and its two target genes, *OsSPX-MFS1* and *OsSPX-MFS2*, provided evidence that they may target different genes compared with Arabidopsis and play a role in phosphate (Pi) metabolism [37]. In Arabidopsis, an SPX domain protein is the *AtNLA* paralog gene although it can't complement the *nla* mutant phenotype [54].

The third category consists of miRNA168 which has been shown to target the *ARGONAUTE1* (*AGO1*) gene, which encodes the RNA slicer enzyme in the miRNA pathway [56]. miR168 and *AGO1* maintain the balance between miRNAs and their targets. Maize miR168 has also been found to be salt stress related and is up-regulated in the salt-tolerant maize inbred line, but down-regulated in the salt-sensitive line [19].

## Methods

### Plant materials, culture, and sampling

The maize inbred line Ye478 was used in this study as it is an important breeding line known to be very sensitive to nitrogen treatment. Seeds of Ye478 were sterilized with 10% (v/v) H_2_O_2_ for 30 min, washed with distilled water, soaked in saturated CaSO_4_ for 6 h, and then germinated at 28°C for 2 d in the dark between two layers of filter paper moistened with saturated CaSO_4_. Seeds 1–2 cm germ were transferred to coarse silica sand to grow at 28°C/22°C during the 14/10 h light/dark cycle. Uniform seedlings with two visible leaves were selected. After discarding the residual endosperms, seedlings were planted in a glass beaker containing 2 L half-strength concentrated solution and changed to full-strength solution the next day. The outside of the glass beakers were covered with a black sheet to ensure that the roots were kept in complete darkness. Glass beakers each containing ten seedlings were maintained in an illumination chamber. Two nitrate concentrations were tested: 4 mM, which represented an optimal nitrate condition (+N) and 0.04 mM, which represented low-nitrate availability (−N) with Ca (NO_3_)_2_·4H_2_O used as the nitrate source. Ca was compensated to 2 mM at −N with CaCl_2_. The other nutrients in solution were (in mmol L^−1^): 0.75 K_2_SO_4_, 0.1 KCl, 0.25 KH_2_PO_4_, 0.65 MgSO_4_.7H_2_O, and 0.2 EDTA-Fe, and in µmol L^−1^, 1.0 MnSO_4_.H_2_O, 1.0 ZnSO_4_.7H_2_O, 0.1 CuSO_4_.5H_2_O, and 0.005 (NH_4_)_6_Mo_7_O_24_.4H_2_O. Air was continuously pumped through the solution (pH 6.0) that was changed every 2 days. For identification of chronic nitrate regulated miRNAs, seedlings were sampled at 15 days for RNA extraction. For transient expression pattern of miRNAs from high to low N, the 15-day-old seedlings were transferred from +N to −N conditions. Then seedlings were sampled at 2, 4, 8, 12, 24, and 48 h after the transfer. The seedlings in +N were sampled as a control. Fresh leaves and root tissues were sampled separately and immediately frozen in liquid nitrogen.

### RNA extraction

Leaf and root tissues harvested in liquid nitrogen were used to extract the RNA immediately. Total RNAs without genome DNA were isolated with Trizol reagent (Invitrogen, Carlsbad, CA, USA). RNA concentration was quantified by using a NANO Drop 2000 spectrophotometer (Thermo Scientific, Wilmington, DE, USA). Each RNA sample was then diluted to 5 ng/µl for miRNA analysis and to 200 ng/µl for target gene and 18S rRNA analyses. RNA samples were stored at −80°C.

### Microarray hybridization

Microarry hybridization system of SmartArray™ was done with the service from CapitalBio Company (Beijing, China). Three replicates and three corresponding dye swap replicates each for leaf and for root using a total of 12 arrays were applied to compare between the high nitrate and chronic low nitrate conditions. The plant miRNA microarrays V2.0 for SmartArray™ from CapitalBio Company were used in this study, and contained 348 well-characterized plant miRNAs from Arabidopsis (*Arabidopsis thaliana*), maize (*Zea mays*), rice (*Oryza sativa*), soybean (*Glycine max*), and other species as noted in the miRBase release 9.1 (http://www.mirbase.org/) [36], 78 predicted miRNAs [34], [35] and various controls (see Table S4). For microarray hybridization, each probe was printed in triplicate using a SmartArray microarrayer (CapitalBio). The labelled RNA was resuspended in 16 µl hybridization solution containing 15% formamide, 0.2% SDS, 3×SSC and 50×Denhardt's. The hybridization was performed at 42°C overnight, and then washed in a solution containing 2×SSC and 0.2% SDS at 42°C for 4 min. After the last washing with 0.2×SSC solution at room temperature for 4 min and spin-drying, slides were scanned using the LuxScan 10K/A scanner (CapitalBio) and raw pixel intensities were extracted with the Lux- Scan 3.0 software. The levels of significance of differentially expressed miRNAs were analyzed using Significance Analysis of Microarrays software (SAM, version 3.02, http://www-stat.stanford.edu/~tibs/SAM/) (Stanford University, USA). The miRNAs with q<0.001 and Ratio >2 or <0.5 were defined as differentially expressed.

The miRNA Arrays system based on Affymetrix GeneChip® was done with the service from ShanghaiBio Company (Shanghai, China). Each sample for root has three biological replications to compare between 15 days high (4 mM) nitrate culture and 2 hr after transient from high (4 mM) to low (0.04 mM) nitrate condition.The Affymetrix GeneChip® miRNA Arrays used in this study contained 6703 miRNA probes from 70 species (redundant miRNAs were included) as described in the miRBase Release 11 (http://www.mirbase.org/) [36]. Procedures were performed following the manufacturer's instructions. About 1 µg total RNA containing low molecular weight (LMW) RNA was pol (A)-tailed and labeled with biotin using FlashTag Biotin for Affymetrix miRNA arrays (Genisphere, Hatfield, PA, USA). 20×Eukaryotic Hybridization Controls (GeneChip® Eukaryotic Hybridization Control Kit, Affymetrix) were incubated at 65°C for 5 min. 21.5 µl biotin-labeled RNA was suspended in 78.5 µl hybridization solution containing 2×Hybridization Mix, deionized formamide, DMSO, 20×Eukaryotic Hybridization Controls, 3 nM Control Oligonucleotide B2 and nuclease-free water, which was incubated at 99°C for 5 min, followed by 45°C for 5 min. The hybridization was performed at 48°C for 16 h. The arrays were then washed and stained with GeneChip® Hybridization Wash and Stain Kit (Affymetrix, Inc.)and then scanned with the GeneChip® Scanner 3000. The Affymetrix© miRNA QC Tool software (Affymetrix, Inc.) was used for data summarization, normalization, and quality control. The miRNAs with *P*<0.05(q<0.001) and fold changes >2.0 or <0.5 were defined as differentially expressed. Three biological replicates were used in all chip hybridization experiments.

### Microarray data formatting and deposition

All microarray data discussed in this publication had been processed into MIAME compliant data and deposited in NCBI's Gene Expression Omnibus [57] and are accessible through GEO Series accession number SuperSeries GSE31492 (http://www.ncbi.nlm.nih.gov/geo/query/acc.cgi?acc=GSE31492), which was composed of and linked to two SubSeries (accessions: GSE31231 and GSE31373).

### Real-time quantitative RT-PCR for mature miRNAs and their target genes

Real-time quantitative RT-PCR for mature miRNAs was performed with stem-loop RT primers specific for mature miRNAs as described [58], [59]. Briefly, 6 nt of the RT primer's 5′ end pairing with the mature miRNA 3′ end was linked to a self-looped 44 bp sequence (5′-GTCGTATCCAGTGCAGGGTCCGAGGTATTCGCACTGGATACGAC-3′) to make up the stem–loop RT-PCR primers, which initiated reverse transcription of the mature miRNA. The reverse transcription product was amplified using a miRNA-specific forward primer and a universal reverse primer, which were designed according to criteria as described [58] with Primer Express 3.0 (Applied Biosystems, Foster City, CA, USA)( Table S5). miRNA target genes in maize were identified according to Zhang et al [20] and also searched in the database maizesequence.org B73 RefGen_v2 released on November, 23 2010 (http://www.maizesequence.org/). The specific primers for real-time RT-PCR on the predicted target genes were designed with the software primer premier 5.0 (PREMIER Biosoft Int., Palo Alto, CA, USA) (Table S6). Maize 18S ribosome RNA (rRNA) was selected as an internal control in real-time quantitative RT-PCR.

For mature miRNAs and their target genes, real time quantitative PCR with SYBR Green I was performed on an Applied Biosystem's 7300 Sequence Detection System (Applied Biosystems, Foster City, CA, USA). Briefly, 20 µl PCR reaction contained about 100 ng cDNA, 9 µl 2.5×RealMasterMix/20×SYBR solution (TianGen, Beijing, China), 250 nM each primer. The reactions were mixed gently and incubated at 94°C for 2 min, followed by 40 cycles of 94°C for 20 s, 60°C for 30 s and 68°C for 30 s. All samples were performed in 3 biological replicates with 2 technical replicates. 18S rRNA was used for each sample as an internal control. The mean and SD are determined from the triplicate samples. The ΔΔCt method [60] was used to determine the expression level differences among samples. For a given time x at low nitrate treatment, ΔΔC_T_ = (C_T_, _miRNA or target, −N,timex_−C_T, 18srRNA, −N, timex_ )−(C_T_, _miRNA or target, +N,timex_ −C_T, 18srRNA, +N, timex_ ) based on equation 9 of ΔΔCt method [60].

## Supporting Information

Table S1 **Chronic (15D ) and transient ( 2 hrs ) low nitrate regulated mature miRNA families and species identified in maize leaves by using the SmartArray microarray platforms and/or stem-loop real time RT- PCR.**
(DOC)

Table S2 **Chronic (15D) and transient (2 hrs) low nitrate regulated mature miRNA families and species identified in maize roots by using the microarray platforms and verified by stem-loop real time reverse transcription PCR.**
(DOC)

Table S3 **The full list of the predicted target genes of identified miRNAs.**
(XLS)

Table S4 **A miRNA micoarray genelist containing 426 miRNAs and some controls used on SmartArray Chip system.**
(XLS)

Table S5 **Primers used for mature miRNAs stem-loop RT-PCR.**
(XLS)

Table S6 **The real time RT-PCR primers for predicted target genes.**
(XLS)
