# Supplementary material for: Genome-Wide Identification of MicroRNAs in Response to Low Nitrate Availability in Maize Leaves and Roots
Source: PLoS One. 2011 Nov 23;6(11):e28009. doi: 10.1371/journal.pone.0028009 (PMC3223196; doi:10.1371/journal.pone.0028009)
Supplement: Table S1 — Chronic (15D ) and transient ( 2 hrs ) low nitrate regulated mature miRNA families and species identified in maize leaves by using the SmartArray microarray platforms and/or stem-loop real time RT- PCR. (DOC) [file pone.0028009.s001.doc]

**Table S1. Chronic (15D ) and transient ( 2hrs ) low nitrate regulated mature miRNA families and species identified in maize leaves by using the SmartArray microarray platforms and/or stem-loop real time RT- PCR.**

| **Family** | **Species** | **Sequence in maize** | **Fold change** | | **Predicted target genes in maize** | **Description of the target genes** |
| --- | --- | --- | --- | --- | --- | --- |
|  |  |  | **Chronic** | **Transient** |  |  |
| miR164 | miR164a-h | UGGAGAAGCAGGGCACGUGCA | **2.13** | 1.05 | GRMZM2G063522, GRMZM2G009892, GRMZM2G008819, GRMZM2G011553, GRMZM2G045927, GRMZM2G114850, GRMZM2G095556 | NAC transcription factor; Cupredoxin; Plastocyanin-like; No apical meristem (NAM) protein; Antifreeze protein, type I; Harpin-induced 1; Exostosin-like; MYB domain transcription factor |
| miR169 | miR169f,g,h | UAGCCAAGGAUGACUUGCCUA | **0.36** | **0.04** | GRMZM2G091964, GRMZM2G000686, GRMZM2G038303, GRMZM2G040349, GRMZM2G078124, GRMZM2G033245, GRMZM2G165488, GRMZM2G008250 | CCAAT-binding transcription factor, subunit B; CCAAT-binding factor, conserved site |
|  | miR169p | UAGCCAAGGAUGACUUGCCGG | **0.37** | **2.42** | GRMZM2G091964, GRMZM2G000686, GRMZM2G038303, GRMZM2G040349, GRMZM2G078124, GRMZM2G033245, GRMZM2G165488, GRMZM2G008250 | CCAAT-binding transcription factor, subunit B; CCAAT-binding factor, conserved site |
|  | miR169i,j,k | UAGCCAAGGAUGACUUGCCUG | **0.37** | **7.53** | GRMZM2G091964, GRMZM2G000686, GRMZM2G038303, GRMZM2G040349, GRMZM2G078124, GRMZM2G033245, GRMZM2G165488, GRMZM2G008250 | CCAAT-binding transcription factor, subunit B;CCAAT-binding factor, conserved site |
|  | miR169d | UAGCCAAGGAGACUGCCUAUG | **0.47** | 1.38 |  |  |
|  | miR169e | UAGCCAAGGAGACUGCCUACG | **0.44** | **0.27** |  |  |
| miR172 | miR172a,b,c,d | AGAAUCUUGAUGAUGCUGCA | **2.31** | **3.32** | GRMZM2G076602, GRMZM2G017847, GRMZM2G160730, GRMZM2G174784, GRMZM2G176175, GRMZM2G700665  GRMZM2G150893 | Actin; Actin/actin-like; AP2-ERF Pathogenesis-related transcriptional factor and ERF; GPR6 orphan receptor; Peroxidase |
|  | miR172e,f | GGAAUCUUGAUGAUGCUGCAU | **2.04** | **3.11** | GRMZM2G076602, GRMZM2G017847, GRMZM2G160730, GRMZM2G174784, GRMZM2G176175, GRMZM2G700665, GRMZM2G150893 | Actin; Actin/actin-like; AP2-ERF Pathogenesis-related transcriptional factor and ERF; GPR6 orphan receptor; Peroxidase |
| miR397 | miR397a,b | UCAUUGAGCGCAGCGUUGAUG | **0.18** | **0.10** | GRMZM2G072808, GRMZM2G419994, GRMZM2G168953 | Multicopper oxidases; Cupredoxin; Laccase; Serine/threonine protein kinase |
| miR398 | miR398b,c | UGUGUUCUCAGGUCGCCCCCG | **0.43** | **0.44** | GRMZM2G068519 |  |
| miR399 | miR399d,j | UGCCAAAGGAGAGCUGCCCUG | **0.24** | 0.78 | GRMZM2G112377, GRMZM2G075870, GRMZM2G326707, GRMZM2G070591, GRMZM2G165734 | Antifreeze protein, type I; Major facilitator superfamily, general substrate transporter; |
| miR408 | miR408 | CUGCACUGCCUCUUCCCUGGC | **0.30** | 0.91 | GRMZM2G066260, GRMZM2G004012, GRMZM2G352678, GRMZM2G023847, GRMZM2G097851, GRMZM2G100121, GRMZM2G104028, GRMZM2G132169, GRMZM2G336337, GRMZM2G384327, GRMZM2G391794, GRMZM2G441768 | Cupredoxin; Plastocyanin-like, Orphan nuclear receptor, NOR1 type ,Leucine-rich repeat, ribonuclease inhibitor subtype, Phosphopantetheine attachment site |
| miR528 | miR528a,b | UGGAAGGGGCAUGCAGAGGAG | **0.20** | 1.29 | GRMZM2G367668, GRMZM2G004106, GRMZM2G039381, GRMZM2G043300, GRMZM2G062069 | Multicopper oxidase, Cupredoxin; Plastocyanin-like; Multi antimicrobial extrusion protein MatE |
| miR827 | miR827 | UUAGAUGACCAUCAACAAACU | **5.35** | **0.42** | GRMZM2G003992, GRMZM2G166976, GRMZM2G344061 | SPX, N-terminal; Tropomyosin; Major facilitator superfamily, general substrate transporter |

Significance Analysis of Microarrays (SAM) and a criterion of fold change >2 (up-regulated) <0.5 (down-regulated) and q value <0.001 were used and shown in bold. In this table, the data of fold changes of chronic treatment was from SmartArray hybridization data. Transient ( 2hrs ) low nitrate regulated mature miRNA families and species in maize leaves were identified by stem-loop real time RT- PCR only. The mature miRNAs that share the same sequences in maize were listed together, for instance, 169n,o,p,q indicated the zma-miRNA169n,o,p,q species. In this table, transient ( 2hrs ) low nitrate regulated mature miRNA families and species identified in maize leaves were identified by stem-loop real time RT- PCR only.
