# Supplementary material for: Genome-Wide Identification of MicroRNAs in Response to Low Nitrate Availability in Maize Leaves and Roots
Source: PLoS One. 2011 Nov 23;6(11):e28009. doi: 10.1371/journal.pone.0028009 (PMC3223196; doi:10.1371/journal.pone.0028009)
Supplement: Table S2 — Chronic (15D) and transient (2 hrs) low nitrate regulated mature miRNA families and species identified in maize roots by using the microarray platforms and verified by stem-loop real time reverse transcription PCR. (DOC) [file pone.0028009.s002.doc]

**Table S2. Chronic (15D) and transient (2hrs) low nitrate regulated mature miRNA families and species identified in maize roots by using the microarray platforms and verified by stem-loop real time reverse transcription PCR.**

| **Family** | **Species** | **Sequence in maize** | **Fold change** | | **Predicted target genes in maize** | **Description of the target genes** |
| --- | --- | --- | --- | --- | --- | --- |
|  |  |  | **Chronic** | **Transient** |  |  |
| miR160 | miR160 a,b,c,d,e,g,h,i,m | UGCCUGGCUCCCUGUAUGCCA | 0.61 | **4.50** | GRMZM2G005284, GRMZM2G081406, GRMZM2G153233, GRMZM2G159399, GRMZM2G369356, GRMZM2G390641 | Auxin response factor; Transcriptional factor B3 |
|  | miR160f | UGCCUGGCUCCCUGUAUGCCG | 1.18 | **3.51** | GRMZM2G005284, GRMZM2G081406, GRMZM2G153233, GRMZM2G159399, GRMZM2G369356, GRMZM2G390641 | Auxin response factor; Transcriptional factor B3 |
| miR167 | miR167a,b,c,d,n,o,p,q,r,s,t | UGAAGCUGCCAGCAUGAUCUA | **0.49** | 1.87 | GRMZM2G028980, GRMZM2G035405, GRMZM2G068328, GRMZM2G073750, GRMZM2G078274, GRMZM2G081158, GRMZM2G089640, GRMZM2G475882 | Auxin response factor;Transcriptional factor B3 |
| miR168 | miR168a,b,c | GUGAAGUGCUUGGGGGAACUC | 0.80 | **3.96** | GRMZM2G039455, GRMZM2G441583 | Argonaute and Dicer protein, PAZ |
| miR169 | miR169i,j,k | UAGCCAAGGAUGACUUGCCUG | **0.33** | **12.52** | GRMZM2G000686, GRMZM2G038303, GRMZM2G040349, GRMZM2G078124, GRMZM2G033245, GRMZM2G165488, GRMZM2G008250 | CCAAT-binding transcription factor, subunit B;CCAAT-binding factor, conserved site |
|  | miR169f,g,h | UAGCCAAGGAUGACUUGCCUA | **0.32** | **9.93** | GRMZM2G000686, GRMZM2G038303, GRMZM2G040349, GRMZM2G078124, GRMZM2G033245, GRMZM2G165488, GRMZM2G008250 | CCAAT-binding transcription factor, subunit B;CCAAT-binding factor, conserved site |
|  | miR169p | UAGCCAAGGAUGACUUGCCGG | **0.25** | **5.81** | GRMZM2G000686, GRMZM2G038303, GRMZM2G040349, GRMZM2G078124, GRMZM2G033245, GRMZM2G165488, GRMZM2G008250 | CCAAT-binding transcription factor, subunit B;CCAAT-binding factor, conserved site |
| miR319 | miR319a,b,c,d,e | UCAUUGAGCGCAGCGUUGAUG | 1.5 | **2.41** | GRMZM2G001279, GRMZM2G180979, GRMZM2G028054, GRMZM2G139688, GRMZM2G089361, GRMZM2G115516, GRMZM2G388987, GRMZM2G412073 | Zinc finger, CCCH-type; Orphan nuclear receptor, NOR1 type; MYB domain transcription factor; TCP Transcription factor; Peptidase C19, ubiquitin carboxyl-terminal hydrolase 2; Zinc finger, MYND-type; |
| miR395 | miR395a,b,e,f,g,h,i,j,n,p, | GUGAAGUGUUUGGGGGAACUC | **0.42** | **4.64** | GRMZM2G042171, GRMZM2G051270, GRMZM2G149952, GRMZM2G122148 | Sulphate anion transporter; ATP-sulfurylase; PUA-like, Methyltransferase-16, putative |
| miR399 | miR399d,j | UGCCAAAGGAGAGCUGCCCUG | **0.24** | **2.20** | GRMZM2G070591, GRMZM2G165734, GRMZM2G075870, GRMZM2G326707, GRMZM2G112377 | Major facilitator superfamily, general substrate transporter; Antifreeze protein, type I; Phosphate permease |
| miR408 | miR408 | CUGCACUGCCUCUUCCCUGGC | **0.42** | 0.97 | GRMZM2G336337, GRMZM2G004012, GRMZM2G352678, GRMZM2G023847, GRMZM2G066260, GRMZM2G097851, GRMZM2G100121, GRMZM2G104028, GRMZM2G132169, GRMZM2G384327, GRMZM2G391794, GRMZM2G441768 | Cupredoxin; Plastocyanin-like, Orphan nuclear receptor, NOR1 type; Leucine-rich repeat, ribonuclease inhibitor subtype, Phosphopantetheine attachment site |
| miR528 | miR528a,b | UGGAAGGGGCAUGCAGAGGAG | **0.38** | 1.34 | GRMZM2G004106, GRMZM2G039381, GRMZM2G043300, GRMZM2G062069, GRMZM2G148937, GRMZM2G169033, GRMZM2G367668, GRMZM2G178741 | Multicopper oxidase, Cupredoxin; Plastocyanin-like; Multi antimicrobial extrusion protein MatE |

Significance Analysis of Microarrays (SAM) and a criterion of fold change >2 (up-regulated) <0.5 (down-regulated) and q value <0.001 were used and shown in bold. The presented data on fold changes of chronic treatment was only from SmartArray hybridization data. The presented data of fold changes of transient treatment, which compared between transient 2hr from high to low and control ( high N) at root, was from Affymetrix miRChip (SBC service) hybridization data. Some of the listed data were verified by real time stem-loop RT PCR on mature miRNAs with three biological replicates (data not shown). The mature miRNAs that share the same sequences in maize were listed together, for instance, 169n,o,p,q indicated the zma-miRNA169n,o,p,q species.
